# Immune Response in Cystic Fibrosis: Interplay between the Host and Microbes

**DOI:** 10.3390/ijms24097766

**Published:** 2023-04-24

**Authors:** Sébastien Boutin, Loïc Guillot

**Affiliations:** 1 Department of Infectious Diseases and Microbiology, University of Lübeck, 23562 Lübeck, Germany; sebastien.boutin@uksh.de; 2 Centre de Recherche Saint-Antoine (CRSA), INSERM UMR S_938, Sorbonne Université, 75012 Paris, France

Cystic fibrosis (CF) is a rare genetic disease caused by genetic variants of the cystic fibrosis transmembrane conductance regulator (CFTR) [1]. This disease is characterized by microbial infections, including bacteria, fungi, and viruses. Studies focusing on the relationship between microbiome and inflammation in people with CF (pwCF) showed that the decrease in diversity is linked to an increase in inflammation. The change in the microbiome from diverse states to almost a mono-specific state is associated with chronic bacterial lung infection, especially by the Gram-negative bacteria *Pseudomonas aeruginosa* [2]. The establishment of such an infection is a major step in the progression of lung disease severity and is known to induce excessive lung inflammation and impair airway epithelial barrier function [1]. In fact, airways constantly exposed to pathogens via respiration have a pivotal role in the triggering and orientation of a protective immune response in the lung. This immune response involves the cells of innate immunity, such as the airway epithelium [3]. In CF, there is evidence that the adaptive immune cell response is also affected [4], although it has been less extensively studied. The use of CFTR-targeted therapies, such as modulators and correctors, has shown promise in partially restoring CFTR function and improving lung function and other clinical outcomes in pwCF. However, the impact of these therapies on cellular immune response and infection remains poorly understood. With the advent of these new disease-modifying treatments for those who have access to them, there is great interest in understanding their impact on the microbiome’s evolution and the mechanisms of microbial infection in CF [5], and a deeper exploration of the host cellular immune response and the microbial mechanisms involved in evading the host or treatment response is necessary to develop new therapeutic strategies.

This collection features one original article and four review articles addressing these topics.

As mentioned above, many studies have focused on the innate immune response to *P. aeruginosa*, including the role of airway epithelium, or phagocytes. An original study about the role of T cells against *P. aeruginosa* is presented by Villeret et al. [6]. Using RAG KO mice that no longer have classical αβ and γδ TCR T lymphocytes and double RAG γC KO mice (lacking T, NK, and ILC cells), they showed that the lymphocytic cell compartment is important for fighting *P. aeruginosa.* They also observed a specific post-transcriptional regulatory mechanism of IL-22 and IL-23 during infection. Furthermore, they elegantly demonstrated that the adenoviral-mediated-overexpression of IL-1, IL-23, and IL7 modifies the immune response, promoting a neutrophil and lymphocytic influx in the lung, preventing mice lethality in wild-type mice and RAG KO and RAGγC mice. The study suggests that using cytokines to manipulate the immune response of the host could be an effective strategy for fighting infections in pwCF. However, additional research is necessary to evaluate the use of cytokines as a therapeutic approach in pwCF.

A first review by Yu et al. [7] discusses the close relationship between inflammation and infection in CF, which together contribute to lung damage and declining lung function. It also highlights the progression of treatments since the discovery of the CFTR gene in 1989, recent advancements in CFTR modulating therapies, and the therapeutic obstacles facing the near and far-off future.

This review is supplemented by a review by Harvey et al. [8] that explains how infections take hold and the microbiome changes in CF, along with the current knowledge of the antimicrobial impact of CFTR modulators on infections.

At the cellular level, a detailed review of the role of macrophages in CF is presented by Jaganathan et al. [9]. The studies carried out were mainly focused on *Burkholderia cenocepacia* and *P. aeruginosa*. Specifically, this review details the mechanisms associated with phagocytosis (initiation, formation, and maturation of the phagosome) as well as the autophagy mechanisms deregulated in CF. The effect of CFTR modulators and other potential therapies on phagocytosis is also presented.

Related to this review, Meoli et al. [10] review the literature on the impact of CFTR-targeted molecules on phagocytes, including not only macrophages but neutrophils. The effect of different CFTR-approved therapies is presented. If at least a partial restoration of macrophage function is achieved with some of the CFTR modulators used, larger studies are still needed to understand their action.
